# A prospective analysis of ureteral stenting during radical cystectomy and ileal conduit urinary diversion: Paediatric feeding tubes versus single‐J stents

**DOI:** 10.1002/bco2.70032

**Published:** 2025-05-26

**Authors:** Jonathan T. Ryan, Tarek Ajami, Adam Williams, Dinno Mendiola, Bruno Nahar, Sanoj Punnen, Chad R. Ritch, Dipen J. Parekh, Mark L. Gonzalgo

**Affiliations:** ^1^ Desai Sethi Urology Institute University of Miami Miller School of Medicine, Sylvester Comprehensive Cancer Center Miami Florida USA; ^2^ Dr. Kiran C. Patel College of Allopathic Medicine (NSU MD) Davie Florida USA

**Keywords:** bladder cancer, ileal conduit diversion, paediatric feeding tubes, postoperative complications, radical cystectomy, single‐J stents, surgical outcomes, ureteral stenting, uretero‐ileal stricture

## Abstract

**Objectives:**

This study compares postoperative outcomes of radical cystectomy (RC) with ileal conduit urinary diversion (ICUD) using paediatric feeding tubes versus single‐J ureteral stents.

**Materials and Methods:**

Patients underwent RC with ICUD for bladder cancer between 2011 and 2018. Prospective preoperative clinical, operative and postoperative data were collected. Postoperative complications including stricture, urine leak, urinary tract infection (UTI) and ileus were compared between patients who received 5‐Fr paediatric feeding tubes or 7‐Fr single‐J ureteral stents during surgery.

**Results:**

Two hundred thirty‐four patients underwent RC with ICUD including 26 with paediatric feeding tubes and 208 with single‐J ureteral stents; 41% had robotic cystectomy, with 36% of these undergoing intracorporeal ICUD. Both groups were comparable in age, gender, kidney function and comorbidities. No significant differences were observed between groups for rates of ileus (20% vs. 34%, *p* = 0.14), urine leak (4% vs. 10%, *p* = 0.3), uretero‐ileal stricture (16% vs. 18%, *p* = 0.7) or overall urinary complications (20% vs. 37%, *p* = 0.12), except for a lower UTI rate in the feeding tube group (4% vs. 23%, *p* = 0.02). Median hospital stay was shorter in the feeding tube group (6 vs. 8 days, *p* = 0.015) with similar readmission rates compared to the stent group (*p* = 0.96).

**Conclusions:**

Using 5‐Fr feeding tubes for ureteral stenting during RC with ICUD is a safe alternative to 7‐Fr single‐J stents, especially for patients with small ureters or delicate anatomy. Stent type showed no significant impact on postoperative urinary complications except for a lower UTI rate with feeding tubes, suggesting comparable overall outcomes between the two stent types.

## 1 INTRODUCTION

Bladder cancer is one of the most common urinary tract malignancies, and about 25% of patients are diagnosed with muscle‐invasive disease for which radical cystectomy with urinary diversion (RCUD) is a standard treatment.^1^, ^2^ RCUD is a complex procedure associated with significant morbidity, and mortality can be approximately 1.7% during the first 90 days.^2^ Additionally, RCUD is associated with a high number of urinary complications including urinary leak, stricture and urinary tract infections (UTI).^3^, ^4^

Ureteral stents are commonly utilized to bridge the uretero‐enteric anastomoses during RCUD, but the overall benefit of ureteral stenting during radical cystectomy (RC) with ileal conduit urinary diversion (ICUD) remains debatable.^3^, ^4^, ^5^ Recent studies have questioned how the duration of stent placement may influence complication rates.^2^ Such a trend toward stentless anastomoses or early removal of ureteral stents is attributed to possible increased risk of infectious complications, bacterial colonization and development of uretero‐ileal strictures.^2^, ^6^ However, stentless RCUD has not been widely adopted among urologists due to the paucity of randomized clinical trials or prospective data. Notably, Mattei et al. in a prospective randomized control trial with 54 patients demonstrated that the ureteral stenting during RCUD is associated with decreased urinary tract dilation, improved bowel function and decreased metabolic acidosis.^7^ On the other hand, Tallman et al. showed in a non‐controlled study the feasibility and safety of omission of ureteral stents during RC with ICUD.^8^

There are multiple stenting options for RC with ICUD including double J stents, single‐J stents and paediatric feeding tubes. Ureteral stents are typically more rigid than feeding tubes and have a retention mechanism such as a pigtail or curl at one or both ends. Additionally, stents have multiple side holes to promote urinary drainage while avoiding obstruction. In contrast, feeding tubes lack a proximal curl and mid or distal side holes, which may result in a higher degree of urine flow around the tubing rather than inside of it. Given the lack of literature in this area, we investigated postoperative outcomes after RC with ICUD comparing the use of 5‐Fr paediatric feeding tubes versus 7‐Fr single‐J ureteral stents to bridge uretero‐enteric anastomoses.

## 2 MATERIAL AND METHODS

We reviewed data collected prospectively from 234 patients who underwent RCUD at a single institution between 2011 and 2018. Only patients who underwent ICUD were included.

Preoperative variables including age, gender, body mass index (BMI), surgical approach (open, robotic extracorporeal or robotic intracorporeal), hypertension, diabetes, smoking status, glomerular filtration rate (GFR), history of prior abdominal surgery or radiation and receipt of neoadjuvant chemotherapy were compared between groups. All RCUD procedures were performed by a total of six urologists.

Two different types of stents were utilized to bridge uretero‐enteric anastomoses during RCUD: 5‐Fr paediatric feeding tubes or 7‐Fr single‐J ureteral stents. The choice of stent type was determined at the surgeon's discretion and based on practice preferences. Patients were compared according to the type of stent used during surgery.

The primary outcome measure was overall urinary complications, which included UTI, urinary leak and ureteral stricture. Postoperative ileus was defined as 4 to 5 days without a bowel movement or the presence of radiological evidence of ileus. Additional outcomes recorded during the initial hospitalization included the need for nasogastric (NG) tube replacement, return to the operating room or an interventional radiology procedure. Postoperative discharge events, such as urgent care or emergency room visit, readmission rate and need for an interventional radiology procedure or corrective operation within 30 days of the initial hospital stay, were also analysed.

Postoperative urinary leak, UTI, intra‐abdominal abscess and sepsis were monitored for up to 30 days following the initial operation. Stricture rates were analysed in relation to the number of months post‐operation in which they occurred and the most recent follow‐up time recorded within a year after surgery. Uretero‐enteric strictures were defined based on imaging findings consistent with stricture, including hydronephrosis confirmed by an abnormal pyelogram, CT scan evidence of stricture or deterioration of renal function. In cases where no deterioration of renal function or urinary infection was present, renal scintigraphy was used to confirm the diagnosis. Intraoperative blood loss, blood transfusion, length of hospital stay (LOS) and use of adjuvant chemotherapy or radiation were also recorded.

Descriptive summaries were presented as frequencies and percentages for categorical variables and as medians with interquartile ranges (IQR) for continuous variables. The chi‐square test was used for categorical variables, while the Mann–Whitney test was applied to continuous variables. Kaplan–Meier analysis was performed to determine cumulative incidence of urinary complication‐free survival. All analyses were two‐sided, with a *p*‐value <0.05 considered statistically significant. Statistical tests were conducted using SPSS version 28.

## 3 RESULTS

Overall, a total of 234 patients underwent RC with ICUD for bladder cancer between 2011 and 2018. Of these, 26 patients (11.1%) received paediatric feeding tubes, while 208 patients (88.9%) received single‐J ureteral stents. The median age was 71 years, with 22.6% of patients being female and 77.4% being male.

Patient characteristics are outlined in Table 1. No significant differences were observed between baseline characteristics of the two groups except for surgical approach (open vs. robotic extracorporeal vs. robotic intracorporeal, *p* < 0.001). Notably, the robotic intracorporeal technique was associated with a significantly higher use of paediatric feeding tubes (72%) compared to single‐J stents (9.6%).

**TABLE 1:** Preoperative patient characteristics.

	Overall	Paediatric feeding tube (*n* = 26)	Single‐J ureteral stent (*n* = 208)	*p*‐Value
Age (median, IQR)	71 (65–77)	75 (66.5–79)	71 (65–77)	0.272
Gender (%)				0.613
Female	53 (22.6)	4 (16)	49 (23.4)	
Male	181 (77.4)	21 (84)	160 (76)	
BMI (median, IQR)	26 (24–30.2)	25.5 (22.7–29.8)	26.7 (24.2–30.3)	0.397
Approach (%)				<0.001
Open	136 (58.1)	6 (24)	130 (62)	
Robotic, extracorporeal UD	60 (25.6)	1 (4)	59 (28)	
Robotic, intracorporeal UD	38 (16.2)	18 (72)	20(9.6)	
Hypertension (%)	136 (58.1)	14 (56)	122 (58)	0.833
Diabetes (%)	56 (24)	5 (20)	51 (24)	0.805
Smoking (%)	181 (77.4)	20 (80)	161 (77)	1
CKD (GFR < 60)	70 (33)	10 (41)	60 (32)	0.362
Prior abdominal surgery (%)	140 (59)	13 (52)	127 (60)	0.39
Prior pelvic radiation (%)	34 (14.7)	4 (16)	30 (14.6)	0.77
Neoadjuvant chemotherapy (%)	53 (22.6)	7 (28)	46 (22)	0.46
Adjuvant chemotherapy (%)	34 (14.5)	2 (8)	32 (15.3)	0.549

Abbreviations: BMI, body mass index; CKD, chronic kidney disease; GFR, glomerular filtration rate; IQR, interquartile ranges; UD, urinary diversion.

Intraoperative and postoperative outcomes are summarized in Table 2. Overall urinary complication rate across both groups was 35% (82 patients). Additionally, overall rates of urine leak and uretero‐ileal stricture were 9% (21 patients) and 17% (43 patients), respectively.

**TABLE 2:** Summary of intraoperative and postoperative outcomes.

	Overall	Paediatric feeding tube	Single‐J ureteral stent	*p*‐Value
Median LOS (median, IQR)	8 (6–11)	6 (5–8)	8 (6–11)	0.015
Blood transfusion (%)	20 (8.6)	2 (8)	18 (9.9)	0.7
Postoperative ileus (%)	77 (33)	5 (20)	72 (34)	0.14
Required NG tube placement (%)	30 (13)	2 (8.7)	28 (13.6)	0.5
Required IR procedure during postoperative period (%)	21 (9)	2 (8.7)	19 (9.1)	0.7
Post‐discharge event				0.96
Urgent care visit (%)	10 (4.2)	1 (4)	9 (4.3)	
Readmission (%)	44 (18.8)	4 (16)	40 (19)	
Postoperative urinary leak (%)	21 (8.9)	1 (4)	20 (9.6)	0.3
Postoperative sepsis (%)	26 (11.1)	1 (4)	25 (12)	0.2
Postoperative UTI (%)	50 (21)	1 (4)	49 (23)	**0.021**
Postoperative uretero‐ileal stricture (%)	43 (17)	4 (16)	39 (18)	0.7
Overall urinary complication (%)	82 (35)	5 (20)	77 (37)	0.12

Abbreviations: IQR, interquartile ranges; IR, interventional radiology; LOS, length of stay; NG, nasogastric; UTI, urinary tract infection.

**FIGURE 1:**
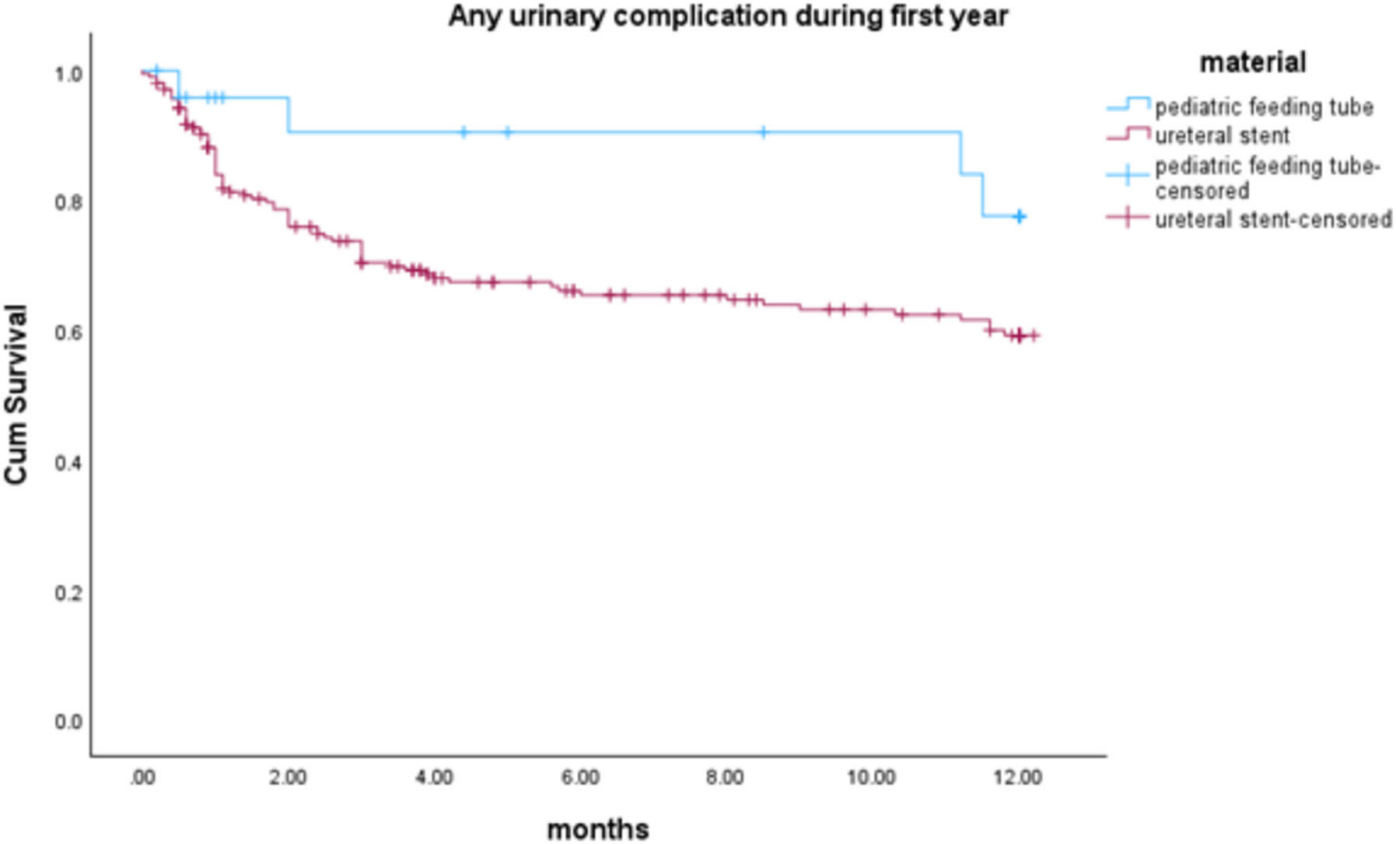
Kaplan–Meier analysis showing urinary complication‐free events during the first year post radical cystectomy (paediatric feeding tube vs. ureteral stent).

There was no statistically significant difference in the rate of overall urinary complications between the paediatric feeding tube group and single‐J ureteral stent group (20% vs. 37%, *p* = 0.12). Kaplan–Meier analysis revealed that the paediatric feeding tube group consistently had a lower, though not statistically significant, rate of urinary complications compared to the ureteral stent group (log‐rank = 0.08), as shown in Figure 1.

No statistically significant differences were observed for postoperative stricture rates between paediatric feeding tubes and ureteral stents (16% vs. 18%, *p* = 0.7) or urinary leak rates (4% vs. 9.6%, *p* = 0.3). Notably, the prevalence of UTIs was lower in the paediatric feeding tube group compared to the ureteral stent group (4% vs. 23%, *p* = 0.021).

Secondary outcomes shown in Table 2 include median LOS, blood transfusion rates, incidence of ileus, percentage requiring NG tube placement, return to the operating room and post‐discharge events. Median LOS was significantly shorter for the paediatric feeding tube group compared to the single‐J ureteral stent group (6 vs. 8 days, *p* = 0.015). Additionally, 30‐day readmission rates were comparable between the two groups with 16% for the paediatric feeding tube group and 19% for the ureteral stent group.

## 4 DISCUSSION

To our knowledge, this is the first study to compare the impact of stent type and size (5‐Fr paediatric feeding tube vs. 7‐Fr ureteral stent) on postoperative outcomes following RC with ICUD. Our analyses revealed no statistically significant differences between paediatric feeding tubes and single‐J ureteral stents in terms of overall postoperative complications, including stricture and urinary leak rates. These findings suggest that the type of stent used to bridge the uretero‐enteric anastomosis (paediatric feeding tube versus single‐J ureteral stent) does not significantly influence postoperative urinary complications. The only notable difference was a significantly lower rate of UTI in the paediatric feeding tube group compared to the single‐J ureteral stent group.

RCUD continues to be associated with a high rate of postoperative complications. Across studies, the rate of post‐cystectomy strictures typically ranges from 10% to 13%.^5^, ^9^, ^10^ In comparison, we observed a stricture rate of 17% in our population. This discrepancy may be due to the variability in how strictures are defined across studies. Similarly, the rate of urinary leak in our study (9%) was higher than rates reported in the literature, which range from 1.9% to 5.5%.^11^ Shah et al., in a large retrospective study, found that stentless patients had a higher rate of urinary leaks but no significant differences in rates of UTI, urinary obstruction or readmissions at 30‐day post‐operation.^12^ Despite these differences, we found no statistically significant difference in stricture rates or urinary leak rates between paediatric feeding tubes and single‐J ureteral stents.

The lack of significant differences in terms of postoperative complications between paediatric feeding tubes and single‐J ureteral stents is noteworthy, since some studies have reported higher postoperative complications in stented versus non‐stented RCUD.^3^ Tallman et al. and Taylor et al. demonstrated that stentless robotic cystectomy with UD is safe and feasible, finding no statistically significant difference between stented and stentless groups in terms of readmission rates or overall complications.^8^, ^13^ However, Tallman et al. observed a lower 30‐day UTI rate in the stentless group.^8^ Lastly Veccia et al. showed in a meta‐analysis of three studies that there was no significant difference between stented and stentless urinary diversion in terms of rates of stricture, 30‐day readmission, UTI and urinary leak.^14^ The utility of intraoperative ureteric stenting during RC with ICUD remains a topic of ongoing debate.^15^

Although single‐J ureteral stents are the most common method for bridging uretero‐enteric anastomoses during RCUD, it remains unclear how this type of stent compares to other commonly used stents in terms of complications. Ibrahim et al. demonstrated that the use of bander stents versus double J ureteric stents during RCUD showed no significant difference between the two groups with respect to stent blockage, stricture or LOS.^16^ Our findings, along with this literature, suggest that alternative stent types, such as paediatric feeding tubes, can be utilized without an increased risk of strictures or urinary complications.

An important limitation of this study is that a higher percentage of the paediatric feeding tube group underwent intracorporeal robotic RC, while a lower percentage underwent open surgery compared to the single‐J ureteral stent group. This difference may have contributed to the lower rates of urinary complications observed in the paediatric feeding tube group. Although the quality of available data comparing intracorporeal and extracorporeal urinary diversion is debated due to a lack of prospective randomized controlled trials, previous literature suggests that complication rates at 30 and 90 days are comparable between the two approaches.^17^, ^18^ Another limitation of this study is the smaller sample size in the paediatric feeding tube group compared to the single‐J stent group. Finally, this study was not a randomized trial designed to isolate these two variables, although we attempted to account for potential confounding factors between the two groups.

## 5 CONCLUSIONS

The use of 5‐Fr paediatric feeding tubes for ureteral stenting during RC with ICUD is a safe alternative to single‐J ureteral stents. Our data suggest that the choice between paediatric feeding tubes and ureteral stents for bridging the uretero‐enteric anastomosis does not significantly affect postoperative urinary complications, except for a reduced incidence of UTI associated with the use of paediatric feeding tubes.

## AUTHOR CONTRIBUTIONS

**Jonathan T. Ryan:** Conceptualization; methodology; data curation; writing—original draft; writing—review and editing. **Tarek Ajami:** Conceptualization; data curation; writing—review and editing. **Dinno Mendiola:** Writing—review and editing. **Adam Williams:** Formal analysis; writing—review and editing. **Bruno Nahar:** Supervision; writing—review and editing. **Sanoj Punnen:** Supervision; writing—review and editing. **Chad R. Ritch:** Supervision; writing—review and editing. **Dipen J. Parekh:** Conceptualization. **Mark L. Gonzalgo:** Conceptualization; methodology; writing—review and editing; supervision.

## CONFLICT OF INTEREST STATEMENT

The authors Jonathan T. Ryan, Tarek Ajami, Adam Williams, Dinno Mendiola, Bruno Nahar, Sanoj Punnen, Chad R. Ritch, Dipen J. Parekh and Mark L. Gonzalgo declare that they have no conflict of interest relevant to this article.

## Data Availability

The datasets generated during and/or analysed during the current study are available from the corresponding author under reasonable request.

## References

[1] Lopez‐Beltran A , Cookson MS , Guercio BJ , Cheng L . Advances in diagnosis and treatment of bladder cancer. BMJ. 2024 Feb;12:e076743. 10.1136/bmj-2023-076743 38346808

[2] Beano H , He J , Hensel C , Worrilow W , Townsend W , Gaston K , et al. Safety of decreasing ureteral stent duration following radical cystectomy. World J Urol. 2021 Feb;39(2):473–479. 10.1007/s00345-020-03191-2 32303901

[3] Donat SM , Tan KS , Jibara G , Dalbagni G , Carlon VA , Sandhu J . Intraoperative ureteral stent use at radical cystectomy is associated with higher 30‐day complication rates. J Urol. 2021 Feb;205(2):483–490. 10.1097/JU.0000000000001329 33238829 PMC8162033

[4] Jarowenko M , Bennett A . Use of single J urinary diversion stents in intestinal urinary diversion. Urology. 1983;22(4):369–370. 6636391 10.1016/0090-4295(83)90411-9

[5] Peng YL , Ning K , Wu ZS , Li ZY , Deng MH , Xiong LB , et al. Ureteral stents cannot decrease the incidence of ureteroileal anastomotic stricture and leakage: a systematic review and meta‐analysis. Int J Surg. 2021 Sep;93:106058. 10.1016/j.ijsu.2021.106058 34416355

[6] Lee CU , Lee JH , Lee DH , Song W . Feasibility and safety of stentless uretero‐intestinal anastomosis in radical cystectomy with ileal orthotopic neobladder. J Clin Med. 2021;10(22):5372. 10.3390/jcm10225372 34830652 PMC8624446

[7] Mattei A , Birkhaeuser FD , Baermann C , Warncke SH , Studer UE . To stent or not to stent perioperatively the ureteroileal anastomosis of ileal orthotopic bladder substitutes and ileal conduits? Results of a prospective randomized trial. J Urol. 2008 Feb;179(2):582–586. 10.1016/j.juro.2007.09.066 18078958

[8] Tallman JE , Vertosick EA , Alam SM , Baky FJ , Donat SM , Pietzak EJ , et al. Perioperative complications and omission of ureteral stents during robot‐assisted radical cystectomy with intracorporeal ileal conduit. J Urol. 2024;10‐1097. 10.1097/JU.0000000000004387 PMC11888893 39666958

[9] Lobo N , Dupré S , Sahai A , Thurairaja R , Khan MS . Getting out of a tight spot: an overview of ureteroenteric anastomotic strictures. Nat Rev Urol. 2016 Aug;13(8):447–455. 10.1038/nrurol.2016.104 27349367

[10] Anderson C , Morgan T , Kappa S , Moore D , Clark PE , Davis R , et al. Ureteroenteric anastomotic strictures after radical cystectomy‐does operative approach matter? J Urol. 2012;189(2):541–547. 23260561 10.1016/j.juro.2012.09.034

[11] Tinoco CL , Lima E . Urinary diversions for radical cystectomy: a review of complications and their management. Mini‐Invasive Surg. 2021;5(28):2574‐1225.

[12] Shah MS , Hochberg AR , Prebay ZJ , Shah YB , Im BH , Simhal RK , et al. Stent vs stentless ileal conduits after radical cystectomy: is there a difference in early postoperative outcomes? Urol Pract. 2024;12(1):139–146. 10.1097/UPJ.0000000000000702 39240682

[13] Taylor Z , Musallam S , Meyer K , Elkhashab I , Thomas B , Snow Z , et al. Tubeless ureteroenteric anastomosis in robot‐assisted radical cystectomy with intracorporeal urinary diversion does not increase the risk of anastomotic stenosis or postoperative complications. J Robot Surg. 2024;18(1):361. 10.1007/s11701-024-02116-0 39367889

[14] Veccia A , Brusa D , Treccani L , Malandra S , Serafin E , Costantino S , et al. Radical cystectomy with stentless urinary diversion: a systematic review and meta‐analysis of comparative studies. Urol Oncol Semin Orig Investig. 2025;43(1):54–60. 10.1016/j.urolonc.2024.06.025 39164149

[15] Yasser O , Harraz A , Barakat T , El‐Halwagy S , Mosbah A , Abol‐Enein H , et al. External stent versus double J drainage in patients with radical cystectomy and orthotopic urinary diversion: a randomized controlled trial. Int J Urol. 2016;23(10):861–865. 27545102 10.1111/iju.13173

[16] Ibrahim M , Nayak A , Patel A , Brodie A , Decaestecker K , Teoh JYC , et al. Comparative study between the use of double J ureteric stents vs bander ureteric stents during robotic‐assisted radical cystectomy with intra corporeal ileal conduit urinary diversion. J Robot Surg. 2024;18(1):5. 10.1007/s11701-023-01766-w 38197975

[17] Tanneru K , Jazayeri SB , Kumar J , Alam MU , Norez D , Nguyen S , et al. Intracorporeal versus extracorporeal urinary diversion following robot‐assisted radical cystectomy: a meta‐analysis, cumulative analysis, and systematic review. J Robot Surg. 2021 Jun;15(3):321–333. 10.1007/s11701-020-01174-4 33222043

[18] Katayama S , Mori K , Pradere B , Mostafaei H , Schuettfort VM , Quhal F , et al. Intracorporeal versus extracorporeal urinary diversion in robot‐assisted radical cystectomy: a systematic review and meta‐analysis. Int J Clin Oncol. 2021 Sep;26(9):1587–1599. 10.1007/s10147-021-01972-2 34146185 PMC8364906

